# Investigation of the Mechanical Behaviors and Damage Mechanism of C/C Composites Impacted by High-Velocity Jets

**DOI:** 10.3390/ma17040963

**Published:** 2024-02-19

**Authors:** Yifan Yue, Bo Wang, Kefei Yan, Renxi Zhao, Chengyu Zhang, Yulong Li

**Affiliations:** 1 School of Aeronautics, Northwestern Polytechnical University, Xi’an 710072, China; 2 Shaanxi Key Laboratory of Impact Dynamics and Engineering Application, Xi’an 710072, China; 3 National Key Laboratory of Strength and Structural Integrity, Xi’an 710065, China; 4 The Sixth Academy of China Aerospace Science and Industry Corporation, Huhehaote 010010, China; yankefei520@163.com; 5 School of Materials Science and Engineering, Northwestern Polytechnical University, Xi’an 710072, China; 6 School of Civil Aviation, Northwestern Polytechnical University, Xi’an 710072, China

**Keywords:** rain erosion, C/C composites, damage mechanism, residual strength

## Abstract

Carbon/Carbon (C/C) composites exhibit excellent mechanical properties at high temperatures, making them widely used in aerospace, such as the leading edges of spaceplane wings and the nose cones of hypersonic aircraft. However, damage caused by rain erosion to C/C composites affects their mechanical properties and poses significant challenges during operational service periods. A jet impingement test platform was employed to conduct single and multiple water-jet erosion tests on three-dimensional orthogonal C/C composite materials and to investigate the residual mechanical properties of the specimens after jet impact. The damage was characterized using optical microscopy, scanning electron microscopy, and X-ray computed tomography. The results showed that the damage types of the C/C composite materials under water-jet impingement included fiber bundle fracturing, delamination, and debonding. The extent of erosion damage was positively correlated with the jet velocity and diameter. The changes in the multi-jet damage indicated a cumulative expansion process, and *z*-directional fiber bundles exhibited superior resistance to jet impact damage propagation. The results of the three-point bending tests showed that the greater the initial impact damage, the lower the residual mechanical properties of the materials, and the residual strength of the specimen suddenly decreased when damage occurred at the back of the specimen.

## 1. Introduction

Carbon/Carbon (C/C) composites are carbon-based composites reinforced with carbon (C) fibers; they exhibit low density, high specific strength, good ablation resistance, and high-temperature mechanical properties [1,2,3,4,5]. They have been widely used in the aerospace field [1,6,7], such as in the nose cones/leading edges of space shuttles, strategic missile warhead tips, engine nozzles, and nose cones of hypersonic aircraft [8,9,10,11,12]. Shi [6] discovered that C/C composites exhibit excellent ablation resistance characteristics. C/C composites are the main ablation materials used in space shuttle nose cones [12]. Scholars have conducted numerous tests on C/C composites in high-temperature and oxidizing environments, and a series of ground and flight tests under hypersonic aircraft programs have been conducted, demonstrating promising application prospects [1,8,13,14].

The U.S. space shuttle Columbia suffered a catastrophic accident due to explosion and disintegration [15]. The accident was attributed to a piece of insulating foam that detached from the spacecraft during launch and struck the C/C composite tile on the left wing, causing a failure of the thermal protection system. Consequently, the high-temperature airflow during reentry caused structural damage, resulting in the accident. As the surface structure of a spacecraft using C/C composites is of utmost importance, the impact resistance performance of the relevant C/C composites requires further research.

With the continuous development of the aerospace field, scholars have systematically studied bird strike problems [16,17,18,19,20], but research on the impact of raindrops and sand on aircraft is insufficient. When an aircraft is in a rainy environment or passes through clouds, its surface is affected by the rain. Because of their high relative velocity, raindrops have a strong impact on the aircraft surface, resulting in surface damage [21,22,23], and the impact process is a mechanical effect. The leading edges of space shuttle wings and nose cones of hypersonic aircraft that use C/C composites also encounter the above-mentioned rain erosion problem during service [24]. Owing to its high relative velocity, the internal structure of an aircraft can be easily damaged, endangering flight safety. Therefore, studying the anti-rain-erosion performance of C/C composites has significant engineering application value.

In 1898, Joukowski [25] used water jets to simulate the impact of rain erosion on solid surfaces and proposed that a high pressure is generated during the impact of liquids on solids. In 1928, Cook [26] proposed the “water hammer pressure” and established its equation to explain the high pressure generated during the impact of liquids on solids. From the 1940s to 1950s, the phenomenon of rain erosion damage to the infrared windows of aircraft attracted widespread interest from scholars [27], and rain erosion problems were subsequently researched experimentally. Existing research on rain erosion has focused on metals [28], glass-fiber-reinforced plastic [27], and polymethyl methacrylate [29,30], whereas research on the rain erosion of today’s widely used composite materials is insufficient.

The impact of rain erosion is essentially a liquid–solid impact process that can be divided into two stages [22]: liquid compression and lateral jet. In the liquid compression stage, at the initial contact of the liquid–solid impact, the shock wave is hindered by the boundary because the velocity at the contact boundary is greater than that of the shock wave inside the liquid, forming an envelope surface. The liquid inside the envelope surface is compressed, generating a high transient pressure, which is the water hammer pressure proposed by Cook [26]. Because of the intense impact compression, the liquid is in a compressible state during this stage. Subsequently, the shock wave reaches the liquid–solid contact boundary, breaks through the boundary, and releases the pressure. The compressed liquid is sprayed along the solid surface to form a lateral jet that enters the lateral jet stage. The lateral jet formed by a high-pressure liquid after release generally has a velocity several times that of the initial water jet and produces strong shear effects.

Current methods of rain erosion protection mainly involve the application of anti-rain-erosion coating technology [31]. However, once the coating is breached, the internal materials are subjected to more severe rain erosion damage. Therefore, enhancing the resistance of internal materials to rain erosion is crucial. Existing research on rain erosion has focused on metals and 2D composites, with limited attention to 3D composites. Therefore, investigating the rain erosion resistance of three-dimensional orthogonal composite materials has important research significance and provides inspiration for the design of rain-erosion-resistant materials. At the same time, due to their outstanding high-temperature performance, C/C composites are increasingly used in high-speed aircraft [1,2,3,4]. However, the high relative velocity between aircraft and raindrops poses serious rain erosion challenges. Therefore, it is of great significance to investigate the rain-erosion-resistance properties of three-dimensional orthogonal C/C composites.

Based on the liquid–solid impact theory, this study conducted high-speed jet impact tests on three-dimensional orthogonal C/C composites, explored their damage mechanism, analyzed their failure modes, and investigated the residual mechanical properties of specimens after impact. The results provide a reference for a deeper understanding of rain erosion theory and the design of rain-erosion-resistant aircraft.

## 2. Materials and Methods

### 2.1. Three-Dimensional Orthogonal C/C Composites

**Figure 1:**
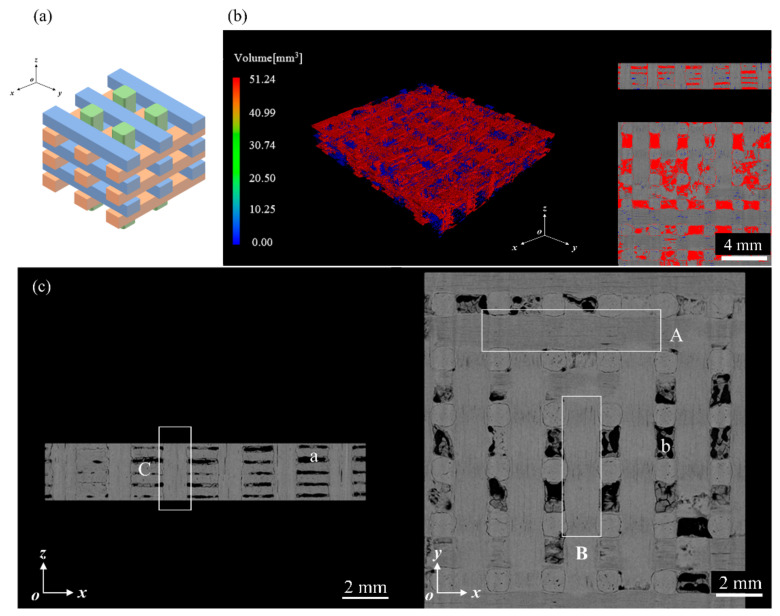
Internal characterization of C/C composite materials: (**a**) three-dimensional orthogonal fiber schematic diagram; (**b**) distribution of material voids; (**c**) X-CT scan of the material structure.

The material studied in this experiment was a composite with a three-dimensional orthogonal structure [32], reinforced with C fibers, and had a matrix composed of pyrolytic carbon. As depicted in Figure 1a, the C fibers were arranged in bundles along the *x*, *y*, and *z* directions in an orthogonal distribution, whereas the matrix material, along with other fillers, occupied the voids.

X-ray computed tomography (X-CT) was used to analyze the structure of the materials. Based on the images depicted in Figure 1c, points A, B, and C correspond to the C fiber bundles in three different directions. The *z*-direction fiber bundles were uniformly dispersed on the *xz*-plane, and the *x*- and *y*-direction fiber bundles were arranged at right angles to each other on the *xy*-plane. The fiber bundle configuration was filled with a pyrolytic carbon matrix; however, some voids were present (Figure 1c). Such defects significantly affect the ability of a material to withstand rain erosion. The interior cavities of the samples were examined using X-CT imaging, as shown in Figure 1b. Determination of the void volume resulted in a material porosity of 10.34%.

**Figure 2:**
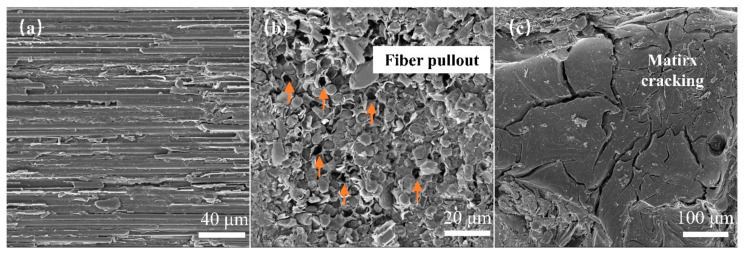
SEM observation of specimen defects: (**a**) fiber bundle in the *x*-direction; (**b**) fiber bundle in the *z*-direction; (**c**) matrix.

Surface defects in the material were observed at the microscopic level via scanning electron microscopy (SEM, TM4000Plus, Hitachi, Tokyo, Japan), with an EHT value of 10.00 kV and a WD value of 30.8 mm. As shown in Figure 2a, the surface of the material exhibited uneven fiber bundle areas with significant roughness. As shown in Figure 2b, a few debonding flaws were observed between the fibers and the matrix in the *z*-directional fiber bundles, causing fiber pull-out phenomena. Additionally, matrix cracking defects were observed in the samples (Figure 2c).

### 2.2. Test Platform

**Figure 3:**
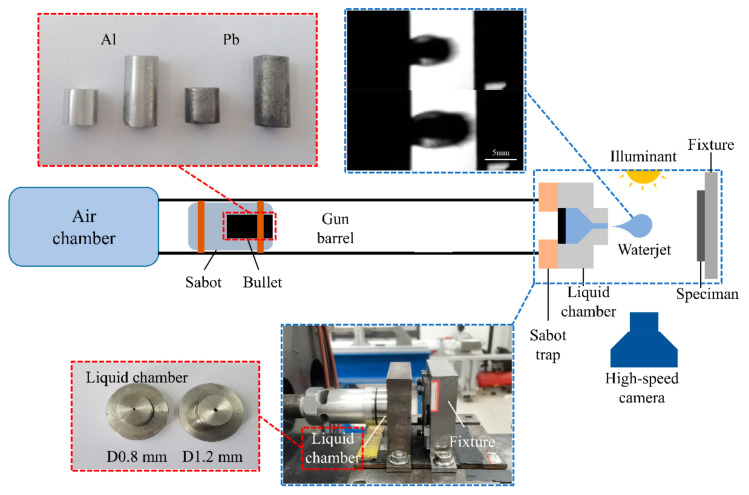
Single-jet impact test platform.

The single-jet impact test platform used in this study was developed based on the single impact jet generator principle of the Cavendish Laboratory’s Single Impact Jet Apparatus (SIJA) and was modified to a single-stage 10 mm light gas gun. As shown in Figure 3, the bullet is accelerated using an air gun, and at the end of the gun barrel, a sabot trap separates the bullet from the sabot. The bullet impacts a rubber plug at the end of the liquid chamber, and under the enormous pressure generated by the impact, high-speed liquid jets are ejected from the nozzle tip. Ultimately, these high-speed liquid jets impact the test specimen, completing the impact process. A high-speed camera (Phantom V711, Vision Research, Wayne, NJ, USA) was used to capture the jet’s morphology, and the jet velocity was measured during the experiment.

Calibration and preliminary tests were performed before the experiments. As shown in Figure 3, the 10 mm displacement jet satisfied the requirements of a rounded leading edge and stable diameter, making it suitable for simulating the impact of rain erosion [20]. The pre-experimental results demonstrated that the diameter of the jet was not affected by the jet velocity. In this study, we assumed that the jet diameter was solely related to the nozzle diameter. For this experiment, we used nozzles with diameters of 0.8 and 1.2 mm to produce jet streams with diameters of 4.7 and 5.8 mm, respectively. Two types of projectiles were used in the experiment to obtain water jets with different velocities and diameters: aluminum and lead. The relevant experimental parameters are listed in Table 1.

Experiments involving various jet velocities, jet diameters, and multiple jet impact events were conducted. The damage was characterized using optical microscopy, SEM, and X-CT to elucidate the mechanisms of material damage. In addition, mechanical performance testing was performed using a micro force material testing machine (Instron MicroTester 5848, Havocan, UK) for three-point bending tests on the C/C composite materials to investigate the impact of the jet impact on the residual mechanical properties of the specimens. The experiments were performed under displacement-controlled loading at a rate of 0.5 mm/min and a sampling frequency of 10 Hz.

## 3. Experimental Results

### 3.1. Single-Jet Impact Tests

Damage characterization analysis was conducted on the test specimens following the impact of a single jet stream at 0°. The selected operating conditions for this analysis were a nozzle diameter of 1.2 mm and jet velocity of 510 m/s, which are typical damage patterns.

**Figure 4:**
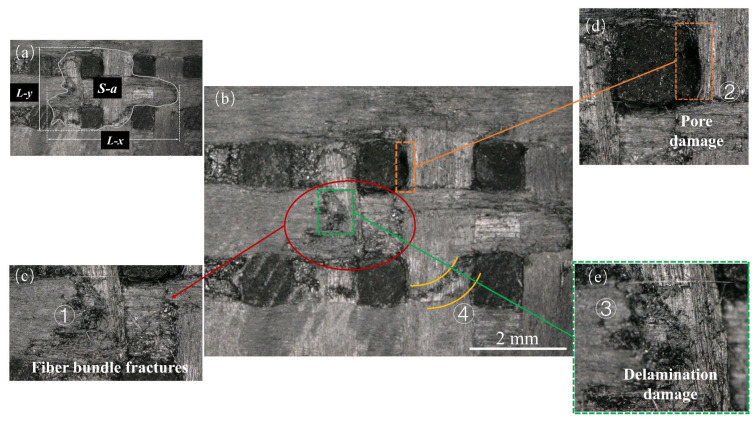
Surface damage under typical operating conditions: (**a**) damage dimension; (**b**) pore damage; (**c**) fiber bundle fractures; (**d**) delamination damage; (**e**) delamination damage.

Figure 4b shows the overall shape of the damage observed in the specimens. The damage was characterized by extensive fiber delamination and matrix detachment, C fiber bundle fractures (Figure 4c ①), delamination damage (Figure 4e ③), matrix detachment leading to *z*-directional fiber bundles debonding and subsequent pore damage (Figure 4d ②), damage along the edges in the form of platforms (Figure 4b ④), partial fiber bundle pull-out, and disordered fiber arrangement after fracturing. The surfaces of the specimens exhibited noticeable craters. Referring to Figure 4a, it can be observed that the damage extension stops on the right side of the *z*-directional fiber bundles. This is attributed to the higher strength of the *z*-directional fiber bundles, giving them enhanced resistance to lateral jet impingement.

Figure 4a shows the quantitative characterization of the surface damage resulting from a high-velocity jet stream impact. *L-x* and *L-y* signify the total lengths of damage in the *x-* and *y*-directions, respectively, and *S-a* indicates the area of damage. This study employed GIMP 2.10.34 to measure both the length and the area of the damage. Table 2 shows the characterization results for the damage caused by the variation of the jet velocities and diameters, which are analyzed further in subsequent sections.

**Figure 5:**
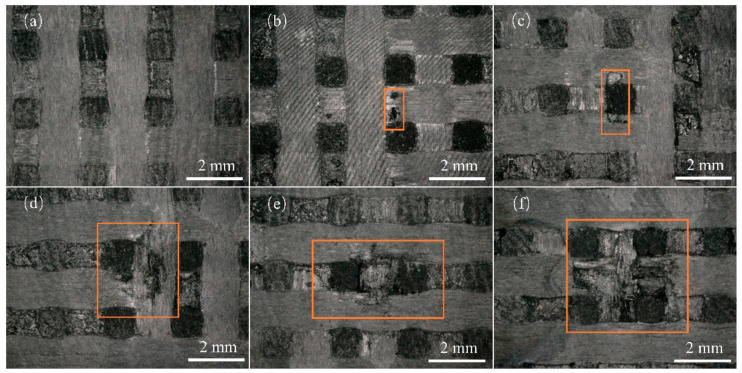
Surface damage caused by different jet velocities for a 0.8 mm nozzle: (**a**) 240 m/s; (**b**) 323 m/s; (**c**) 380 m/s; (**d**) 450 m/s; (**e**) 510 m/s; (**f**) 570 m/s.

Figure 5 shows the surface damage of the specimens at different jet velocities observed using an optical microscope with a nozzle diameter of 0.8 mm. Under the impact of a jet with a velocity of 240 m/s, the surface of the specimen appeared smooth and undamaged (Figure 5a). As the jet velocity increased to 323 m/s, some fibers within the transverse fiber bundle on the specimen surface were partially severed (Figure 5b); however, the surface remained relatively flat without significant depressions. The remaining specimens did not exhibit any visible damage. Therefore, in this experiment, the threshold velocity for single impact damage with a nozzle diameter of 0.8 mm was considered to be 323 m/s. As the jet velocity continued to increase, the extent of the damage became more pronounced, with complete fracture of the fiber bundles, detachment of the C matrix, debonding between the fibers and matrix, and severe splitting of the fiber bundles being observed. At a jet velocity of 450 m/s, delamination damage occurred in the specimens, and there was a light-colored damage platform at the edge of the damage zone.

**Figure 6:**
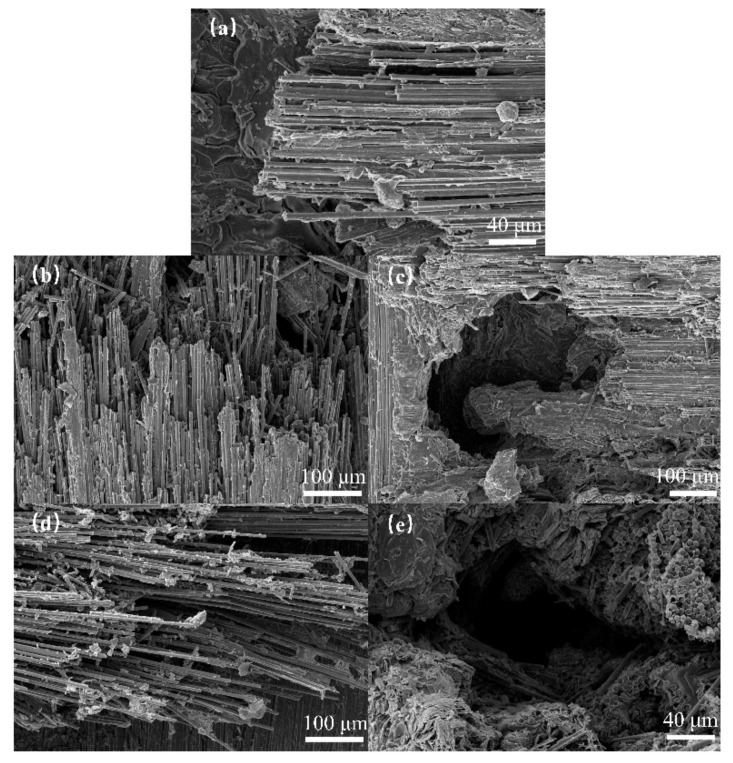
SEM characterization of damage for different jet velocities with a 0.8 mm nozzle: (**a**) 380 m/s; (**b**,**c**) 450 m/s; (**d**,**e**) 570 m/s.

Figure 6 shows the surface damage of the specimens observed using SEM after impact with jet velocities of 380, 450, and 570 m/s using a nozzle diameter of 0.8 mm. The images show that as the jet velocity increased, the extent of damage to the fiber bundle on the specimen surface increased. Partial fiber fractures gradually progressed to complete fiber bundle fractures, with more disordered fiber bundle breakage. In addition, matrix-fiber debonding became more pronounced, with entire sections of the fiber bundles detaching and peeling off. Delamination and splitting damage occurred in *z*-directional fiber bundles, resulting in the formation of voids and holes within the surrounding fiber bundles. Figure 5 and Figure 6 show that as the jet velocity increased, the severity of the debonding and delamination damage, fiber fracture, and delamination damage increased. This indicated that both the water hammer pressure and the lateral jet stream action were amplified.

**Figure 7:**
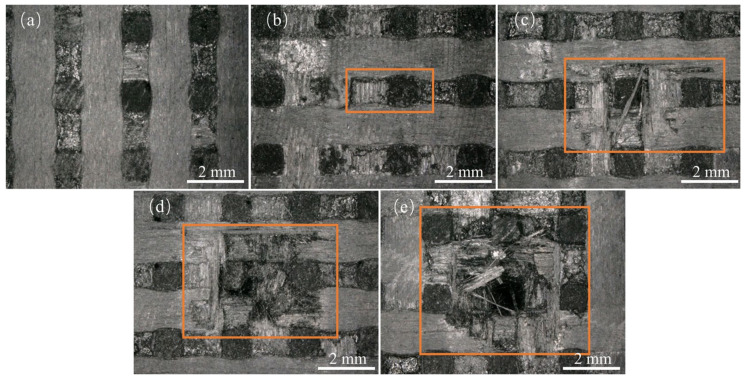
Surface damage caused by different jet velocities for a 1.2 mm nozzle: (**a**) 300 m/s; (**b**) 360 m/s; (**c**) 450 m/s; (**d**) 510 m/s; (**e**) 570 m/s.

**Figure 8:**
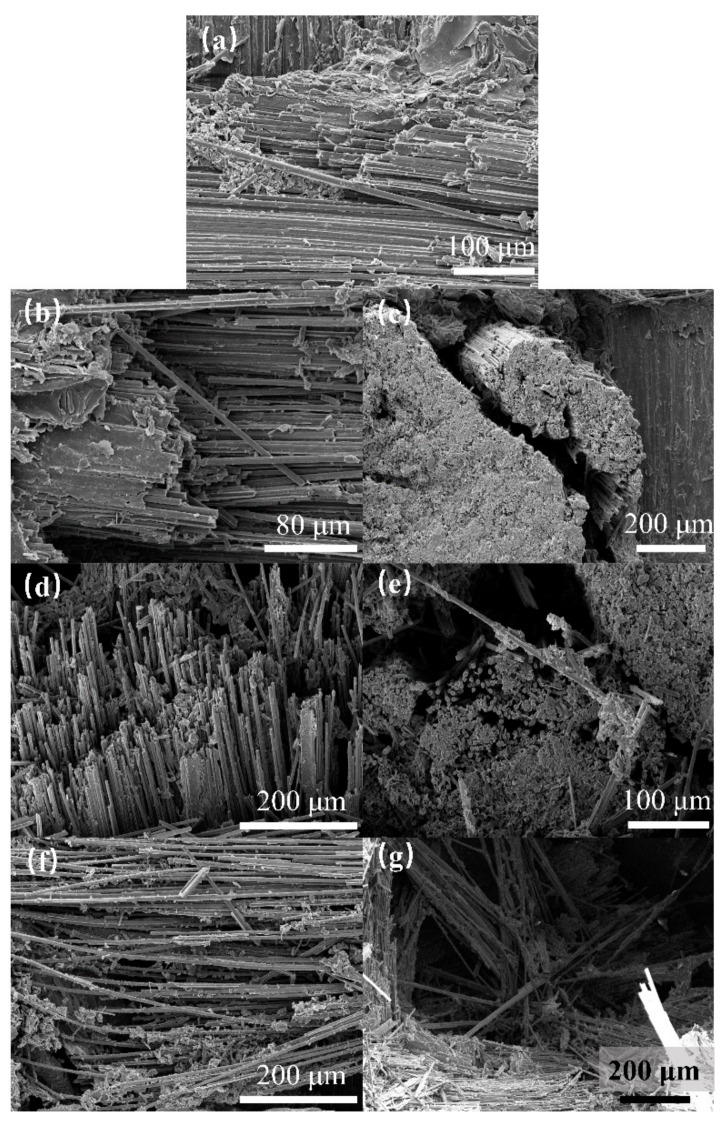
SEM characterization of damage for different jet velocities with a 1.2 mm nozzle: (**a**) 360 m/s; (**b**,**c**) 450 m/s; (**d**,**e**) 510 m/s; (**f**,**g**) 570 m/s.

Figure 7 and Figure 8 depict the surface damage of the specimens for different jet velocities, as observed using optical microscopy and SEM, respectively, using a nozzle diameter of 1.2 mm. Based on image observations, the threshold velocity for single-impact damage with a nozzle diameter of 1.2 mm was determined to be 300 m/s. Similar to the 0.8 mm nozzle diameter, the severity of debonding and delamination damage, fiber fracture, and delamination damage increased with increasing jet velocity. When the jet velocity reached 570 m/s, the damage at the center of the specimen was close to penetration, indicating that the damage depth also increased with increasing jet velocity.

**Figure 9:**
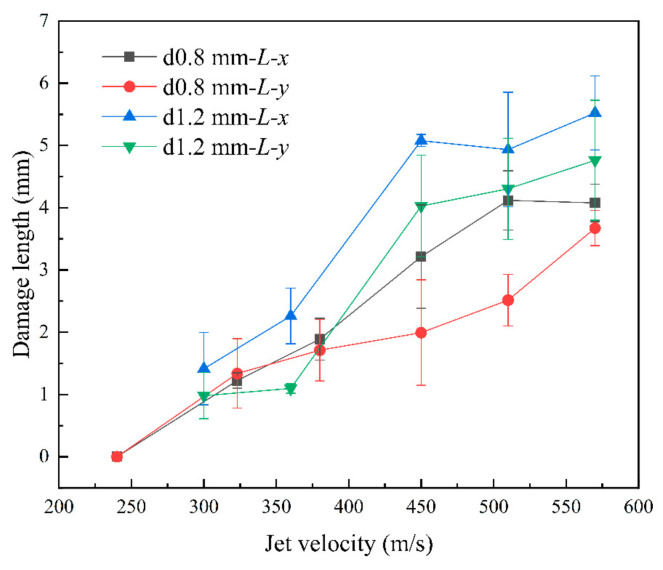
Length of damage at different jet velocities.

**Figure 10:**
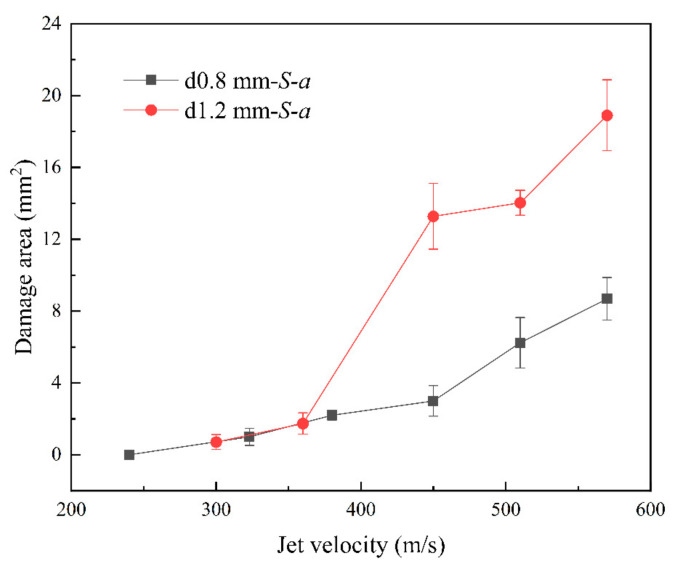
Area of damage at different jet velocities.

Figure 9 and Figure 10 show that as the jet velocity increased, the maximum damage lengths in both directions and the damage area increased. The delamination damage became more pronounced, indicating a significant enhancement in the shearing effect of the lateral jet stream.

By comparing *L-x* and *L-y*, we observed a difference between the transverse and longitudinal damage lengths. The width and spacing of the *x*- and *y*-directional fiber bundles were measured using GIMP 2.10.34, which revealed that the width of the *x*-directional fiber bundles was smaller than that of the *y*-directional fiber bundles. Here, width refers to the overall width of the fiber bundle, representing the fiber content of each directional bundle. In addition, the density of the *x*-directional fibers was lower, indicating a slightly lower strength in the *x*-direction than in the *y*-direction. This was consistent with the result that the *x*-directional damage length was slightly greater than the *y*-directional damage length after impact. This reflected the differences between the *x*- and *y*-directional fiber bundles, indicating the anisotropic behavior of the specimen in the *x-* and *y*-directions.

**Figure 11:**
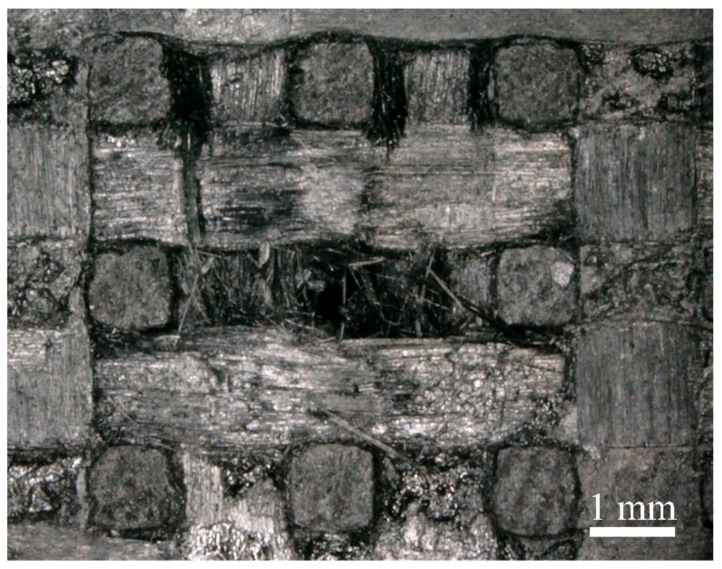
Damage to the backside of the specimen.

Please note, that at a jet velocity of 570 m/s for the two different jet diameters, as shown in Figure 11, fiber fractures were observed on the rear side of the specimens after the jet impact. For a nozzle diameter of 1.2 mm, some specimens experienced penetration damage.

### 3.2. Multi-Jet Impact Tests

To better observe the resistance effect of the *z*-directional fiber bundles on impact damage, multi-jet impact tests were conducted under specific conditions where the nozzle diameter was 0.8 mm and the jet velocity was 510 m/s, ensuring that no damage occurred on the rear surface of the specimens after impact, indicating negligible effects of reflected waves. This magnified observation was intended to highlight the limitation imposed by *z*-directional fiber bundles. The specimens were secured to the test rig to withstand multiple successive single-jet impacts and to simulate the effects of multiple jet impacts.

**Figure 12:**
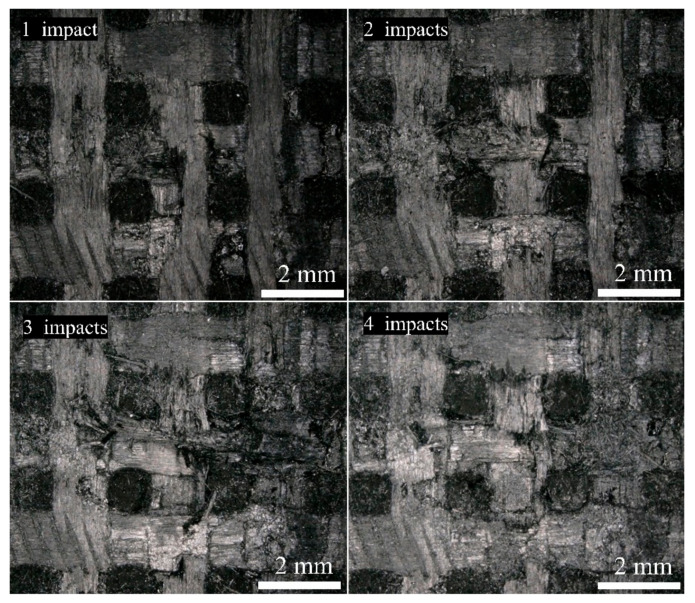
Surface damage evolution after multiple jet impacts.

Figure 12 shows the damaged images of the same specimen after one–four jet impacts. As the number of jet impacts increased, the fiber bundle fracturing and peeling became more severe, resulting in an increased area of surface damage. In addition, the delamination intensified, and the damage depth increased. The presence of inherent voids, cracks, and other defects in the specimen significantly influenced the expansion of the damage. As the number of impacts increased, the damage was more likely to connect with the defects and continue to expand, resulting in an increased area of damage in both the surface and depth directions. This indicated that multiple jet impacts involved a cumulative damage process.

**Figure 13:**
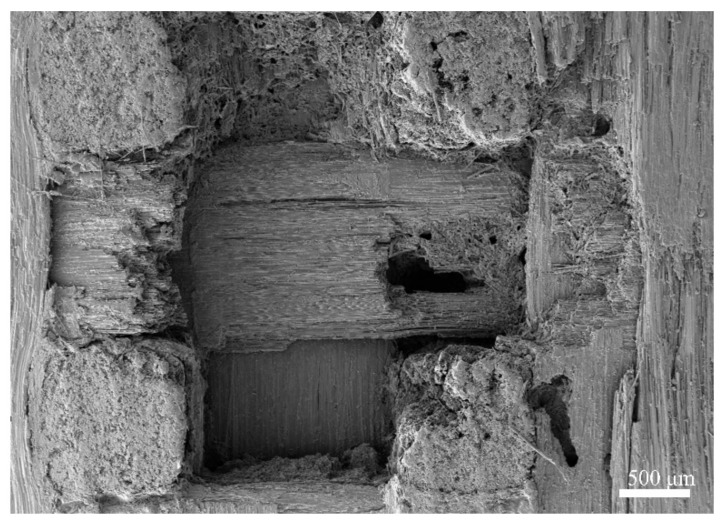
Surface damage of the specimen after four jet impacts.

Figure 13 shows the damage characterization of the specimen after four jet impacts. We observed that the internal fiber bundle fractured within the specimen. Under multiple jet impacts, the fiber bundles experienced progressive damage layer-by-layer, with a consistent damage pattern, increased damage depth, and increased delamination. It is worth noting that the extent of damage to the *z*-directional fiber bundles at the impact site is relatively minimal, exhibiting mainly interfacial delamination and fiber fracture at the boundary. Damage propagates along the central direction of the *z*-directional fiber bundles with minimal expansion compared to the other directions.

Table 3 lists the damage dimensions and damage depths of the specimens subjected to different numbers of jet impacts. The surface damage area of the specimens increased with the number of jet impacts. The damage expansion rate slowed down between impacts 3 and 4. As the number of impacts increased, delamination and crack damage occurred after multiple impacts connected the internal defects, and the lateral jet damage primarily affected the interior of the specimen. In addition, the lateral jet underwent morphological degradation as it passed through the defects and cracks during impact. When it reached a certain surface area, its damage capacity weakened and was insufficient to cause further significant surface damage. With an increasing number of jet impacts, the damage depth gradually increased, indicating a cumulative and progressive expansion of the damage in the C/C composite material.

### 3.3. Residual Mechanical Properties after Jet Impact

**Figure 14:**
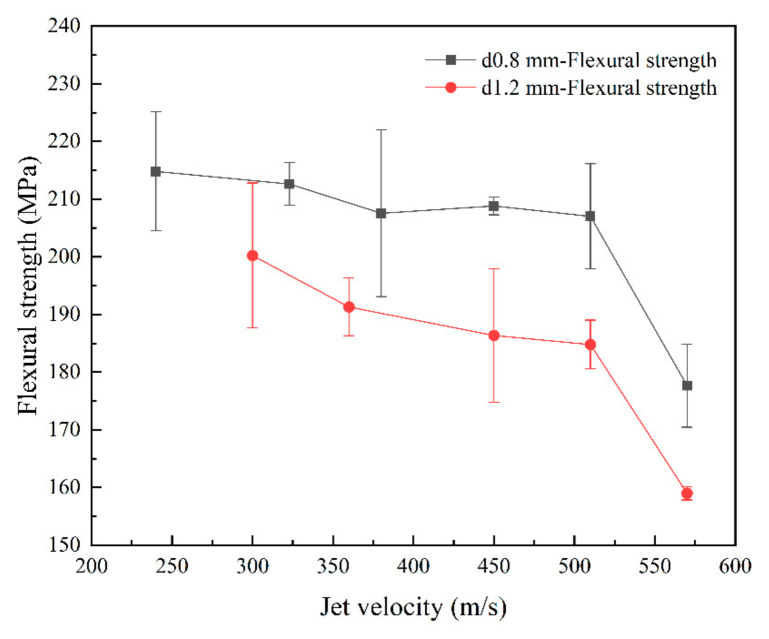
Flexural strength of specimens after different jet impingements.

In order to investigate the impact of jet on the mechanical properties of the material, three-point bending tests were carried out on the specimens subjected to jet impact. The strength results of single-jet impact specimens (Figure 14) show that as the jet velocity and jet diameter increased, the flexural strength of the specimens gradually decreased. The flexural strength of the specimens decreased with increasing jet velocity, but it decreased relatively slowly below 510 m/s, exhibiting a nearly linear trend. When the jet velocity reached 570 m/s, there was a significant decrease in the bending strength of the specimens. In addition, the images show that as the diameter of the jet increased, the flexural strength of the specimens decreased. Experiments revealed that as both the jet diameter and velocity increased, the residual flexural strength of the specimens decreased.

**Figure 15:**
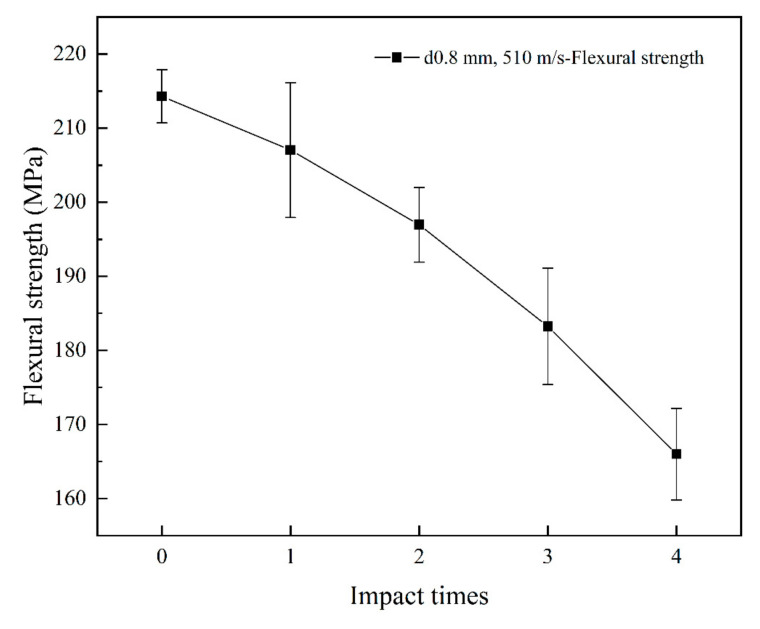
Flexural strength of specimens after different jet impingement cycles.

The strength results of multi-jet impact specimens indicated that the flexural strength of the specimens gradually decreased as the number of jet impacts increased. After four jet impacts, the flexural strength decreased by more than 20%. The experimental results are presented in Figure 15.

## 4. Discussion

### 4.1. Damage Mechanism of Jet Impact

**Figure 16:**
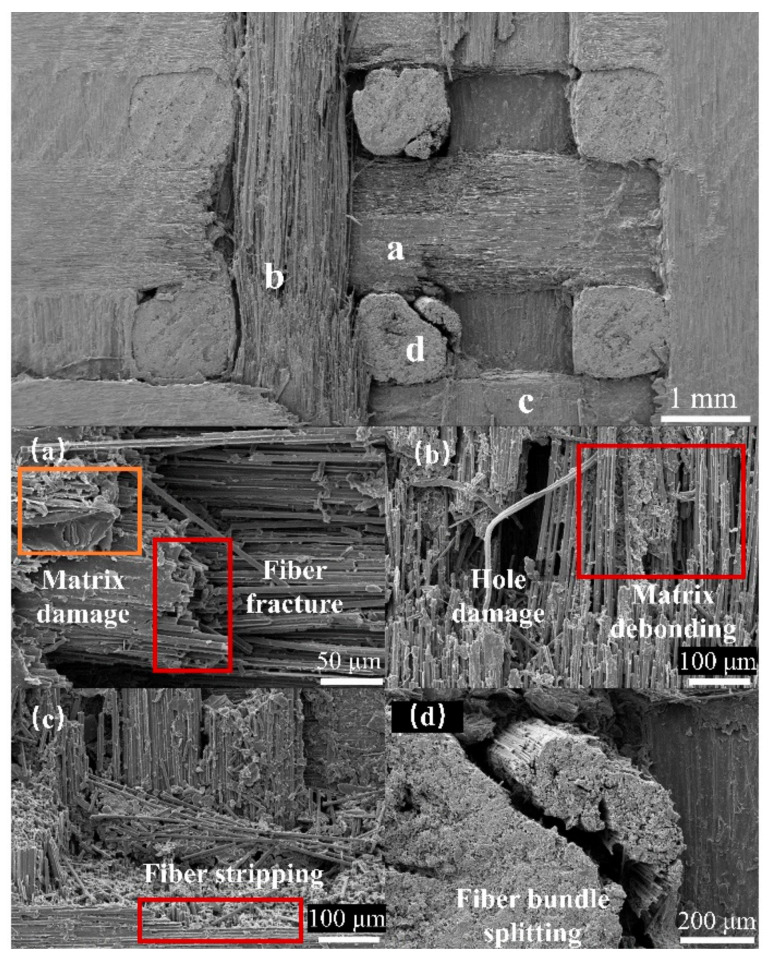
Typical damage characterization using SEM: (**a**) matrix damage and fiber fracture; (**b**) hole damage and matrix debonding; (**c**) fiber stripping; (**d**) fiber bundle splitting.

Damage characterization was performed on the specimens after single-jet impingement to elucidate the damage mechanisms of the three-dimensional orthogonal C/C composite materials. Fiber bundle fracturing, debonding, and delamination are the predominant types of damage observed and can be attributed to the combined effects of the water hammer pressure and high-speed lateral jet streams [22,23]. The damage platforms at the specimen edges were attributed to the lateral jet streams. Figure 16 shows the microdamage at a nozzle diameter of 1.2 mm and a jet velocity of 510 m/s. The surface damage primarily consisted of fiber bundle fracturing and delamination, fiber–matrix debonding and peeling, pore damage, and matrix damage. During the liquid compression phase, the high water hammer pressure [25,26] exerted by the jet on the specimen surface caused initial damage, such as fiber bundle fracture, matrix debonding, and detachment. In addition, a significant water hammer pressure near the impact center resulted in the propagation of downwardly extending cracks. In the second impact phase, the high-speed lateral jet penetrated the subsurface cracks and preexisting surface defects, resulting in extensive peeling damage and, in severe cases, delamination. When the jet velocity was high, debonding and peeling occurred at the boundaries of the *z*-direction fiber bundles, resulting in surface pore damage.

**Figure 17:**
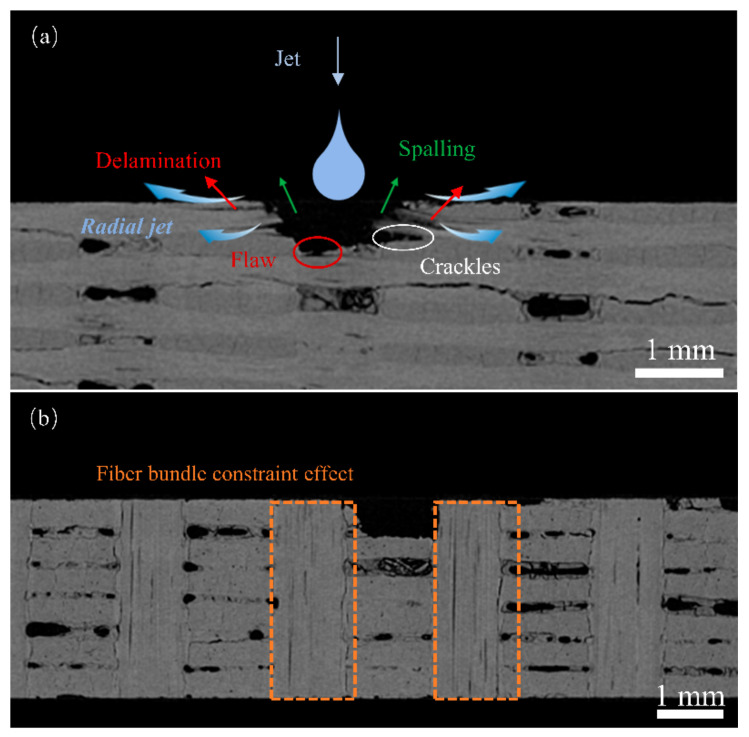
Internal damage: (**a**) damage mechanism; (**b**) square-shaped pit damage.

Figure 17a shows the internal damage to the specimen. During the liquid compression phase, the water hammer pressure caused inclined cracks to extend downward near the impact center, resulting in severe debonding between the fibers and the matrix. Additionally, the initial damage was linked to pre-existing internal defects. In the lateral jet impact phase, a high-speed lateral jet penetrated the subsurface cracks caused by the water hammer pressure and discontinuities of pre-existing surface defects. The lateral jet velocity was several times the initial jet velocity, causing further damage propagation at the sites of initial damage and preexisting defects under the impact of the lateral jet, resulting in deeper layer damage. From the X-CT images of the cross-section of the specimen, we observed that debonding damage occurred at the intersection of the fiber bundles, forming internal cracks.

In contrast to 2D woven materials [23], equally dense *z*-directional C fiber bundles in the jet impact direction. When the impact center was near the *z*-directional fiber bundles, the internal damage differed from that shown in Figure 17a. As shown in Figure 17b, the damage to the inner pit occurred as relatively flat squares. Due to the *z*-directional fiber bundles being perpendicular to the lateral jet, the jet impact had a minimal effect on them, resulting in a limited damage range imposed by the *z*-directional fiber bundles on both sides during the impact. This led to the formation of square pits.

At a jet velocity of 570 m/s for two different jet diameters, damage was observed on the rear side of the specimens after the jet impact. This was attributed to the generation of stress waves (compression waves) on the surface of the specimen owing to the jet impact, which propagated towards the backside of the specimen. The stress waves reaching the rear side of the specimen were reflected and resulted in the formation of tensile waves. Under the combined action of compression and tensile waves, a significant tensile stress was exerted on the backside of the specimen, resulting in damage, such as fiber fracture and delamination.

When the jet velocity was low, the presence of many voids in the C/C composite material enabled the absorption of energy from the stress waves, thereby reducing the intensity of stress waves. Therefore, at velocities below 510 m/s, the effect of the compression waves propagating to the back side of the specimen and interacting with the reflected tensile waves was minimal, and no damage occurred on the back side of the specimen.

### 4.2. Effects of Jet Velocity and Diameter

The damage threshold velocities for the two jets were determined by comparing the experimental results for two different nozzle diameters. The damage threshold velocities for single-jet impacts with diameter nozzles of 0.8 and 1.2 mm were 323 and 300 m/s, respectively. As the nozzle diameter increased, the damage threshold velocity decreased, indicating a positive correlation between the jet size and specimen damage.

Figure 9 and Figure 10 show that at the same jet velocity, the damage length and area were both greater for the 1.2 mm nozzle diameter than for the 0.8 mm nozzle diameter. Moreover, based on the slope of the curves, for the nozzle diameter of 1.2 mm, as the jet velocity increased, the increase in the damage length and area became more significant, indicating a faster rate of damage expansion. This further confirmed the positive correlation between jet size and the extent of damage.

Figure 9 and Figure 10 show that as the jet velocity increased, the extent of damage increased. However, for a nozzle diameter of 1.2 mm, at velocities greater than 450 m/s, the rate of increase in the surface damage area decreased significantly. During the jetting phase, the lateral jet encountered the defects and initial damage caused by water hammer pressure, as well as the process leading to shear damage, this disrupted its morphology and dissipated impact energy. As a result, damage could not form on a larger scale.

**Figure 18:**
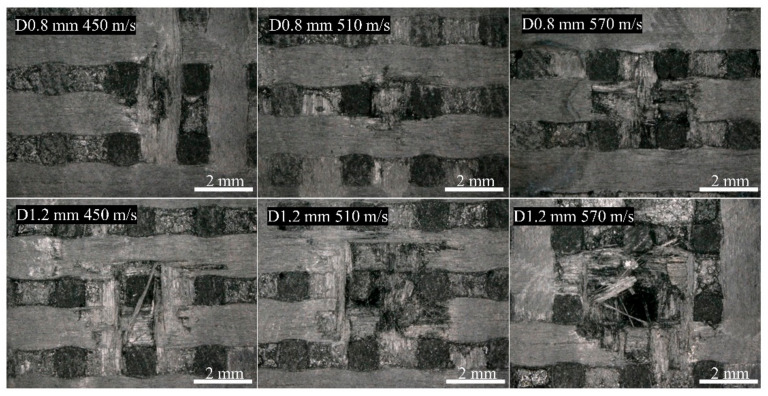
Surface damage at different jet diameters.

Figure 18 shows that at the same jet velocity, the extent of damage was greater with a nozzle diameter of 1.2 mm than 0.8 mm, with apparent aggravation of the delamination damage. During the liquid compression phase, the water hammer pressure was higher for the 1.2 mm condition at the same jet velocity, resulting in more severe initial damage, including a larger range of fiber fracture and debonding. In addition, the lateral jet in the 1.2 mm condition had a higher water flow rate and stronger shearing action, penetrating the initial damage and material defects with greater intensity during the jetting phase, resulting in a larger range of delamination and peeling damage. When the jet velocity reached 570 m/s, the specimen for the 1.2 mm nozzle diameter almost experienced penetrating damage, indicating a greater depth of damage than for the 0.8 mm diameter. Because of the increased initial damage, the initial damage to the specimen was more likely to be connected to pre-existing voids and defects in the material, resulting in a greater depth of initial damage. During the lateral jetting phase, the depth of crack invasion beneath the surface increased, accompanied by increased water flow and shearing action, ultimately resulting in a greater depth of shear-induced damage.

### 4.3. Z-Directional Fiber Bundles Damage Resistance

**Figure 19:**
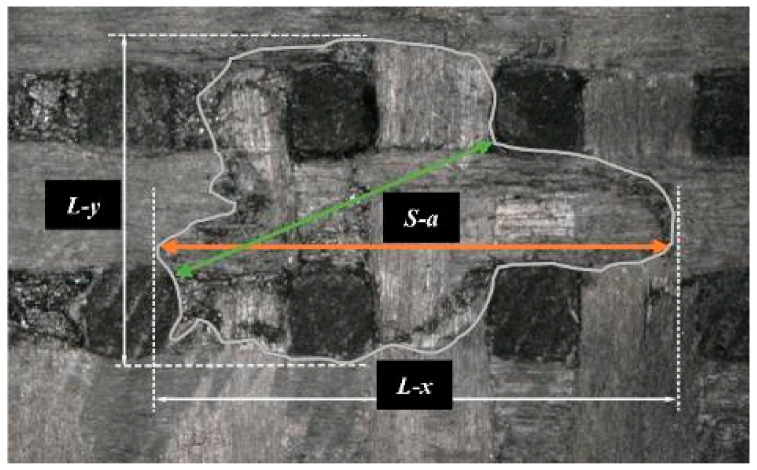
Damage dimension in different directions.

The damage image (Figure 19), under typical conditions, revealed that the initial damage extends from the center [22], with the length of damage propagation in the *z*-directional fiber bundles being significantly smaller than in other directions, due to the woven structure of the material.

**Figure 20:**
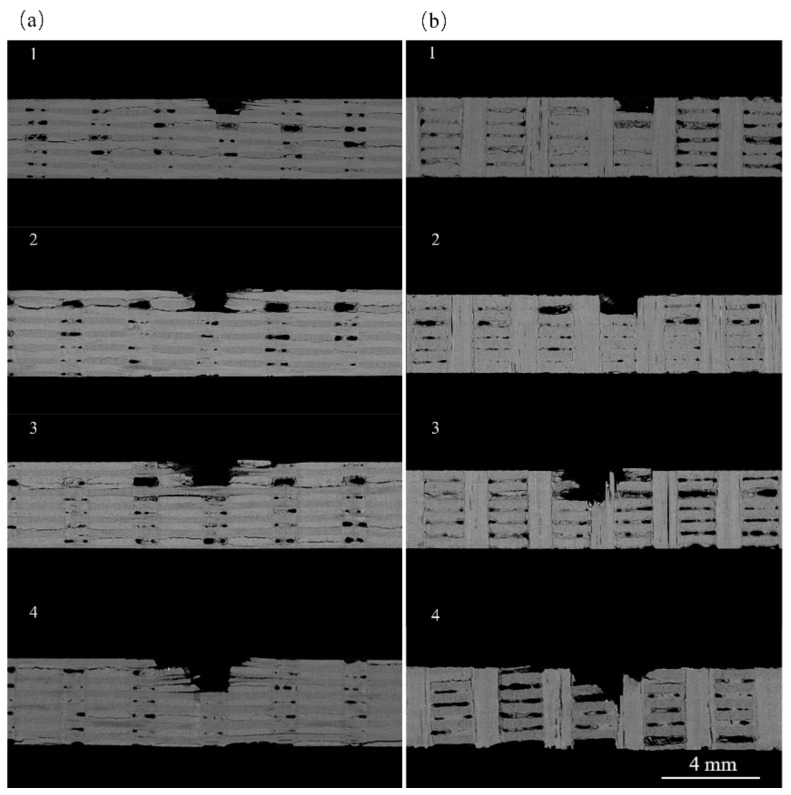
Internal damage of the specimen under different jet impact numbers: (**a**) *xz*-plane; (**b**) *yz*-plane.

**Figure 21:**
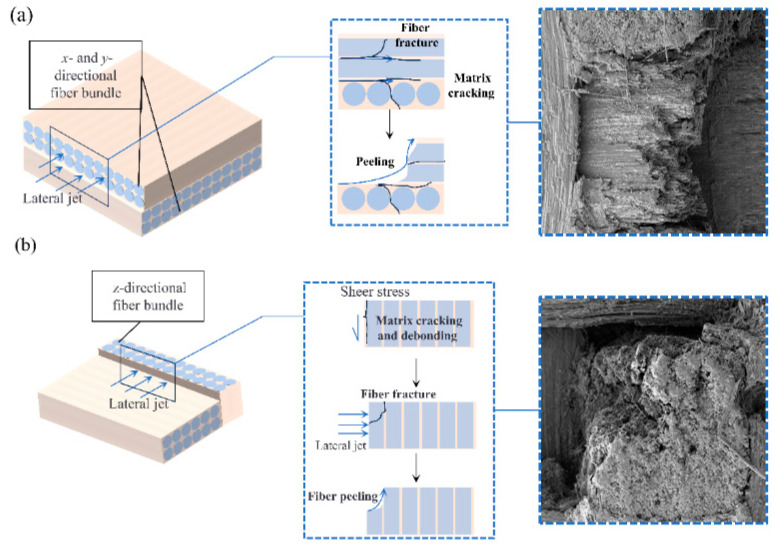
Damage mechanisms at different fiber bundle locations: (**a**) in-plane fiber bundles; (**b**) *z*-directional fiber bundle.

The analysis of specimens after four impact events assessed the resistance of *z*-directional fiber bundles to damage propagation. Figure 20a shows the internal damage within the *yz*-plane for jet impacts 1–4, and the damage extension in this direction is not constrained by the *z*-directional fiber bundles. The predominant damage modes of the specimens were fiber bundle fracturing, peeling, and delamination. As illustrated in Figure 21a, during the first jet impact, surface fiber bundle fracturing and peeling occurred accompanied by subsurface cracks, resulting in a small inverted trapezoidal pit. Internal defects in the specimen connected with the impact damage, enabled the lateral jet to penetrate a larger area during subsequent impacts, resulting in increased internal damage. Under the combined effects of the water hammer pressure and lateral jet, the damage depth gradually increased, causing deeper layers of fiber bundles to experience fracture and peeling damage.

Figure 20b shows the image of internal damage in the *xz*-direction, exhibiting an overall “square” pattern of damage. As illustrated in Figure 21b, when the lateral jet passes through the *z*-directional fiber bundles, the interface is identified as a weaker region. Accordingly, the lateral jet initiates shear action on the interface between the *z*-directional fiber bundles and the matrix. Cracks propagate along the interface, resulting in interface debonding and matrix delamination. Subsequently, the *z*-directional fiber bundles experience a vertical impact. This is due to the vertical arrangement of the *z*-directional fiber bundles in the lateral jet spray direction, resulting in minimal in-plane cracks and defects along the lateral jet spray direction. Therefore, this hindered the shearing action of the lateral jet spray and restricted damage propagation.

**Figure 22:**
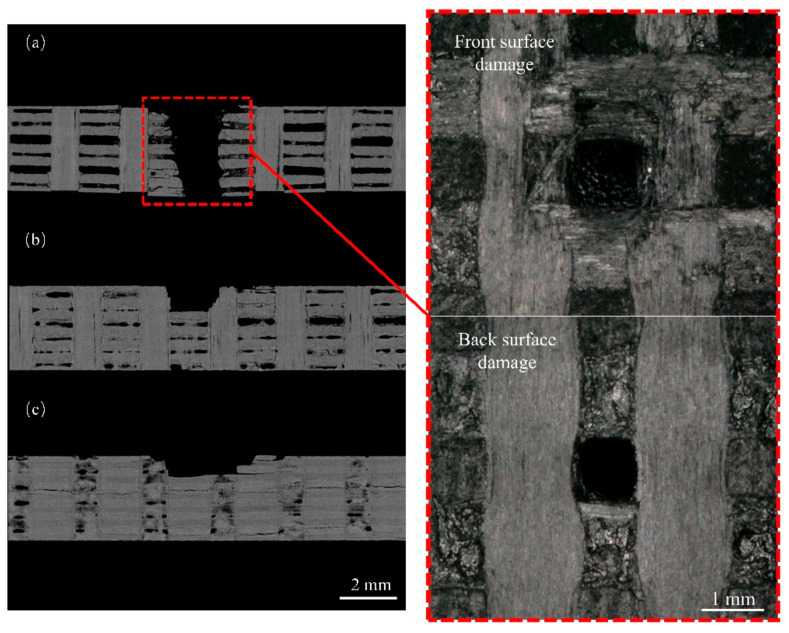
Internal damage characterization of four impinging jet impacts at different center locations: (**a**) *z*-directional fiber bundle; (**b**) *y*-directional fiber bundle; (**c**) intersection of *xy*-fiber bundles.

By comparing Figure 22b,c, it can be observed that the internal damage length in (b) is significantly lower than in (c), indicating that the *z*-directional fiber bundles exhibit a pronounced inhibitory effect on in-plane damage.

Note that under the four jet impacts, some specimens experienced penetration damage. As shown in Figure 22a, when the impinging jet center was located in the *z*-directional fiber bundle, the main damage modes were debonding and penetration of the *z*-directional fiber bundle. Owing to the poor adhesion between the C matrix of the specimen material and the fiber bundle, the interface between the *z*-directional fiber bundle and the other fiber bundles was relatively weak. After multiple impacts, the C matrix severely detached, making it prone to debonding and penetration when the impinging jet center was on the *z*-directional fiber bundle. Observation of the front and back damage of the specimen (Figure 22a) revealed square fracture surfaces on the front side, indicating fracturing and delamination damage to the plane fiber bundle caused by the lateral impinging jet. On the back side, a neat square aperture was observed, indicating the complete penetration of the entire *z*-directional fiber bundle and significant debonding damage.

Based on the above analysis, it can be concluded that *z*-directional fiber bundles exhibit a significant limiting effect on in-plane damage propagation. Although the weak interface may cause damage to the bonding between the *z*-directional fiber bundles and the matrix, the subsequent impact direction of the lateral jet is perpendicular to the fiber bundles. In addition, *z*-directional fiber bundles are thicker and exhibit a superior resistance to lateral jet impingement compared to the planar fiber bundles and matrix. Therefore, the lateral jet passing through the *z*-directional fiber bundles absorbs a significant portion of the impact energy, resulting in a significant limitation on damage propagation.

### 4.4. Mechanism of Decreased Residual Mechanical Properties

The significant decrease in the bending strength of specimens under high-speed impact is attributed to a change in the damage mode of specimens under high-velocity impact. When the impact velocity of the specimen reached 570 m/s, the damage mode of the specimen changed, with damage occurring on the back side of the specimen (Figure 11), and a significant increase in the number of damaged fibers within the specimen. During the three-point bending process, due to the presence of initial damage on the rear surface of the specimen, a more severe stress concentration occurs at the internal damage location. Consequently, the ability of the fibers to withstand shear loads, and that of the matrix to withstand compressive loads, decreased. This resulted in a significant deterioration in the residual mechanical properties of the specimens.

**Figure 23:**
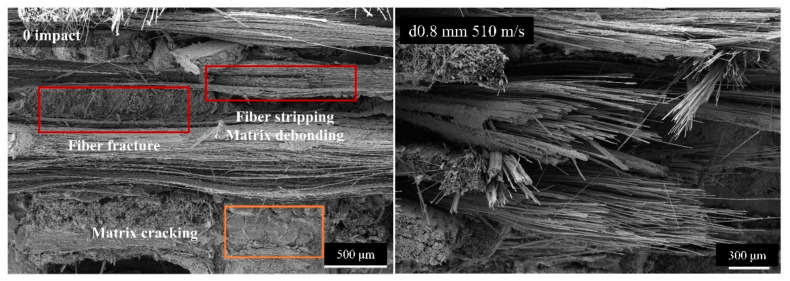
Characterization of bending fracture surfaces using SEM.

As shown in Figure 23, SEM examination of the fracture surface of the specimen revealed three types of damage during bending: matrix cracking, fiber–matrix interface debonding and splitting, and fiber fracturing. After the jet impact, the types of bending damage in the specimens remained unchanged, indicating that the failure mode during bending did not change. A comparison of the fracture morphologies revealed that the post-jet impact specimens exhibited a more disordered arrangement of fractured fiber bundles and increased debonding damage. An analysis and comparison suggested that this was the result of the initial damage caused by the intensification and extension of the impact. The initial jet impact resulted in the fracturing of the surface fiber bundles and the formation of cracks. During the bending process, the damage was more likely to spread to locations with lower resistance, where fiber bundles were fractured and cracks had occurred. This resulted in more severe splitting, debonding, and other forms of damage, ultimately resulting in a decrease in the residual flexural strength.

**Figure 24:**
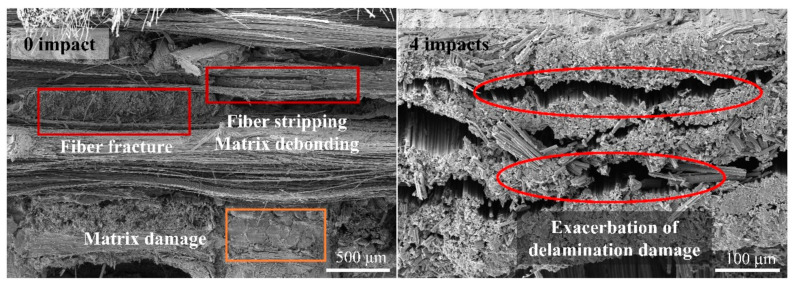
Bending fracture surface of specimens after multiple jet impacts.

As shown in Figure 24, the damage pattern during the bending process was the same as that observed in the specimens after a single-drop jet impact. A comparison of the fracture morphologies showed that, as the number of jet impacts increased, debonding, splitting, and other forms of damage intensified, resulting in a decrease in the residual flexural strength.

## 5. Conclusions

Jet impingement tests were performed on three-dimensional orthogonal C/C composite materials under different operating conditions, and the residual mechanical properties of the materials after jet impingement were studied. The experimental results revealed the following patterns in the water-jet impingement damage of the C/C composites:

(1) Under the action of single-jet impingement, the damage types of C/C composite materials included fiber bundle fracturing, delamination, and debonding. The damage threshold velocity for a single-jet impingement was 323 m/s when the nozzle diameter was 0.8 mm and 300 m/s when the nozzle diameter was 1.2 mm. The extent of damage was positively correlated with the jet velocity and diameter.
(2) *Z*-directional fiber bundles in three-dimensional orthogonal C/C composites exhibit a significant control effect on jet impact damage. Through multi-jet impact tests, the limitation of *z*-directional fiber bundles on in-plane damage propagation becomes more pronounced. The results showed that fiber bundles oriented perpendicular to the jet impact direction have superior impact resistance, highlighting their effectiveness in limiting in-plane damage expansion.
(3) The experimental results indicated that a larger jet impact damage corresponded to lower residual mechanical properties of the material. At higher impact velocities, the manifestation of the stress wave action initiates a shift in the damage mode of the specimen, whereby reflected tensile waves induce damage to the rear of the specimen. The three-point bending tests resulted in an increase in stress concentration, ultimately leading to a marked deterioration of the residual mechanical properties.

## Figures and Tables

**Table 1:** Summary of experimental values.

Test Parameters	
Nozzle orifice diameter	0.8 mm, 1.2 mm
Jet diameter	4.7 mm, 5.8 mm
Chamber pressure	0.2–0.7 MPa
Jet velocity	240–570 m/s
**Target Parameters**	
Dimension	96 mm × 16 mm
Thickness	3 mm

**Table 2:** Damage results of composite material specimens subjected to water-jet impact at different velocities and diameters.

Nozzle Orifice Diamter/mm	Jet Velocity/ms^−1^	*L-x*/mm	*L-y*/mm	*S-a*/mm^2^
0.8	240	0.000	0.000	0.000
0.8	323	1.224	1.338	1.003
0.8	380	1.890	1.712	2.204
0.8	450	3.214	1.993	2.998
0.8	510	4.117	2.516	6.237
0.8	570	4.077	3.673	8.684
1.2	300	1.415	0.981	0.710
1.2	360	2.262	1.097	1.737
1.2	450	5.078	4.027	13.276
1.2	510	4.933	4.304	14.032
1.2	570	5.522	4.762	18.897

**Table 3:** Characterization of specimen damage after multiple jet impacts.

Impact Times	*L-x*/mm	*L-y*/mm	*S-a*/mm^2^	Depth/mm
1	4.117	2.516	6.237	0.712
2	4.123	2.754	7.896	0.729
3	4.619	4.220	12.049	1.01
4	4.827	4.580	14.106	1.659

## Data Availability

The data used to support the findings of this study are available from the corresponding author upon request.
